# The Origin of Malarial Parasites in Orangutans

**DOI:** 10.1371/journal.pone.0034990

**Published:** 2012-04-20

**Authors:** M. Andreína Pacheco, Michael J. C. Reid, Michael A. Schillaci, Carl A. Lowenberger, Biruté M. F. Galdikas, Lisa Jones-Engel, Ananias A. Escalante

**Affiliations:** 1 Center for Evolutionary Medicine and Informatics, the Biodesign Institute, Arizona State University, Tempe, Arizona, United States of America; 2 Department of Anthropology, University of Toronto, Toronto, Ontario, Canada; 3 Department of Social Sciences, University of Toronto Scarborough, Toronto, Ontario, Canada; 4 Department of Biological Sciences, Simon Fraser University, Burnaby, British Columbia, Canada; 5 Department of Archaeology, Simon Fraser University, Burnaby, British Columbia, Canada; 6 National Primate Research Center, University of Washington, Seattle, Washington, United States of America; 7 School of Life Sciences, Arizona State University, Tempe, Arizona, United States of America; Museum National d'Histoire Naturelle, France

## Abstract

**Background:**

Recent findings of *Plasmodium* in African apes have changed our perspectives on the evolution of malarial parasites in hominids. However, phylogenetic analyses of primate malarias are still missing information from Southeast Asian apes. In this study, we report molecular data for a malaria parasite lineage found in orangutans.

**Methodology/Principal Findings:**

We screened twenty-four blood samples from *Pongo pygmaeus* (Kalimantan, Indonesia) for *Plasmodium* parasites by PCR. For all the malaria positive orangutan samples, parasite mitochondrial genomes (mtDNA) and two antigens: merozoite surface protein 1 42 kDa (MSP-1_42_) and circumsporozoite protein gene (CSP) were amplified, cloned, and sequenced. Fifteen orangutans tested positive and yielded 5 distinct mitochondrial haplotypes not previously found. The haplotypes detected exhibited low genetic divergence among them, indicating that they belong to one species. We report phylogenetic analyses using mitochondrial genomes, MSP-1_42_ and CSP. We found that the orangutan malaria parasite lineage was part of a monophyletic group that includes all the known non-human primate malaria parasites found in Southeast Asia; specifically, it shares a recent common ancestor with *P. inui* (a macaque parasite) and *P. hylobati* (a gibbon parasite) suggesting that this lineage originated as a result of a host switch. The genetic diversity of MSP-1_42_ in orangutans seems to be under negative selection. This result is similar to previous findings in non-human primate malarias closely related to *P. vivax*. As has been previously observed in the other *Plasmodium* species found in non-human primates, the CSP shows high polymorphism in the number of repeats. However, it has clearly distinctive motifs from those previously found in other malarial parasites.

**Conclusion:**

The evidence available from Asian apes indicates that these parasites originated independently from those found in Africa, likely as the result of host switches from other non-human primates.

## Introduction

Malarias, including those capable of infecting humans, are caused by a diverse group of parasitic protozoa belonging to the genus *Plasmodium*. Species from this genus are found in a broad range of vertebrate hosts including reptiles, birds, and mammals. Molecular phylogenetic studies have shown that host switches are relatively common in these parasites, as evidenced by the limited host specificity observed in those species parasitic to mammals and birds [1]–[13]. As an example, the four *Plasmodium* species commonly found in humans do not form a monophyletic group but rather are part of two distinct clades of primate malarias. One clade that includes three human malarial parasites (*P. vivax*, *P. ovale*, and *P. malariae*) together with several *Plasmodium* species found in non-human primates [1], [2], [8], [9], [10], and a second clade that includes *P. falciparum* and several lineages recently detected in African apes [1], [2], [3], [4], [5], [6], [7], [13].

Despite a staggering diversity of non-human primate malarial parasites in Southeast Asia when compared to those currently known in Africa and South America, molecular phylogenetic studies have shown that the origin of these species is relatively recent, between 4 to 7 million years ago [2], [8]–[11], [13]–[15]. Our current understanding is that non-human primate malarias were introduced into Southeast Asia by a non-Hominidae primate where they underwent an explosive species radiation [2], [8], [14]. There were several host switches as part of that process, including the one that allowed the colonization of humans by the lineage that originated the parasite *P. vivax* [2],[8],[14]. Thus, the argument follows, host switching events and parasite species radiation took place relatively late in the evolution of these primate malarias. Yet, no evidence for *falciparum*-like parasite has been found in Asian non-human primates including apes [16]–[18]. Hence, the available data support an “Asian model” for the origin of *P. vivax* and an “African model” for the origin of *P. falciparum*.

Although the Asian origin of *P. vivax* from a non-human primate is widely accepted, it is still a work in progress. New data could be consistent or rather change our perspective on the origin of this parasite. As an example, lineages of African *P. vivax* have been found in chimpanzees and humans [5], [19], [20]. These findings of African *P. vivax* (human or ape), however, are posterior introductions into Africa from Asia as indicated by molecular data [19], [20]. Indeed, this parasite (*P. vivax*) seems to have a higher prevalence in the African human populations than previously thought [19], [20]. Thus, finding these *P. vivax* lineages in Africa is still consistent with the prevailing Asian model.

However, the phylogenetic studies sustaining such widely accepted scenario for the origin of *P. vivax* are still missing data from most Southeast Asian apes [2]–[4], [6]–[8], [10], [13]–[15]. Given the absence of suitable samples, a possibility that has not been systematically explored is whether ancestors of Southeast Asian apes could have introduced non-human primate parasites into Asia. Such possibility seems plausible given that orangutans and gibbons naturally harbor malarial parasites [16]–[18]. After such hypothetical ape driven introduction into Asia, malaria parasites could have undergone a secondary radiation into macaques and other non-ape primates found in Southeast Asia.

The ancestors of the modern orangutans, for example, diverged from other members of hominidae between 16–14 Mya in Eurasia [21], [22] and colonized Southeast Asia as early as 9–7 Mya [23], much earlier than the radiation of macaques in Asia [24]. Hence, we could hypothesize that if the lineages leading to the extant orangutan malaria parasites were introduced by ancestral apes, then they would appear as stem lineages in the phylogeny of Asian non-human primate malarias and even change our current understanding of the *P. vivax* origin.

In this study, we estimate molecular phylogenies including a *Plasmodium* lineage found in orangutans from Kalimantan, Indonesia. The samples used in this investigation were previously studied using a short fragment of the 18S rRNA gene [25]. The use of this gene in *Plasmodium* phylogenies is problematic given its slow rate and semi-convergent evolution patterns [26]. Indeed, this gene does not allow differentiating among closely related species [27]. Nevertheless, this study suggested that orangutans from Kalimantan may have been infected by human and macaque malarias in a recent time, at an ecological time scale or only few generations ago [25]. This assertion was challenged by a re-analyses of the data [28] showing that the parasite found may have not been the human parasite *P. vivax*, though it was closely related to macaque parasites and to *P. hylobati*, a parasite found in gibbons. Unfortunately, the phylogenetic signal was not sufficient to resolve the relative position of the orangutan lineage in the *Plasmodium* phylogeny so it is possible that the amplified 18S rRNA fragment represents a stem parasite from which the other non-human primate lineages and *P. vivax* originated. The morphological information was insufficient to identify these parasites as a previously known species.

In addition to nearly complete mitochondrial genomes from the orangutan *Plasmodium* lineage, we also report data from genes encoding two antigens: the merozoite surface protein 1 42 kDa (MSP-1_42_) fragment and the gene encoding the circumsporozoite protein (CSP). Overall, we found that this lineage infecting orangutans is derived from other non-human primate malarias and it is closely related to *P. hylobati* (the gibbon parasite) and *P. inui*, a parasite of wide distribution found in non-hominid primates, mostly macaques suggesting a host-switch. Nevertheless, using relaxed molecular clock methods, we found no evidence indicating that such a host switch took place at an ecological time scale. Indeed, our investigation indicates that this lineage originated approximately 3 million years ago.

## Results

### Phylogenetic analyses

A total of 24 orangutan blood samples from the “Orangutan Care Center and Quarantine (OCC&Q)” in Kalimantan were processed [25]. We found 15 orangutans positive by PCR using primers targeting the mitochondrial Cytochrome b (see materials and methods). We generated sequences of the parasite's mitochondrial genomes (mtDNA) for 11 PCR positive samples. We found, five *Plasmodium* haplotypes in our sample of orangutans that have not been describe previously for non-human primates. However, the average distance among the orangutan *Plasmodium* haplotypes (0.0031±0.0003) is relatively small and similar to the average distance previously found within well characterized *Plasmodium* species (e.g. *P. chabaudi*, *P. cynomolgi*, *P. knowlesi*, and *P. vivax*, see Table 1). Thus, the average genetic distance among the mitochondrial haplotypes suggests that they belong to one species so it will be further referred as genetic polymorphism. Whereas positive blood smears were available [25], they were not of sufficient quality to identify these parasites as belonging to any of the previously described orangutan *Plasmodium* species, *P. pitheci* and *P. sylvaticum* [16], [17].

**Table 1:** Genetic divergences among different *Plasmodium* species.

		Genetic distance (d ± Std Err.)				
Species	n	COXI	COXIII	CYTB	COXI+CYTB	Complete mtDNA
*P. chabaudi chabaudi*	7	0.0011±0.0005	0.0017±0.0009	0.0005±0.0003	0.0009±0.0003	0.0010±0.0003
*P. chabaudi adami*	2	0.0035±0.0016	0.0051±0.0025	0.0035±0.0017	0.0035±0.0013	0.0030±0.0007
*P. ch. chabaudi - P.ch. adami*	7 vs. 2	0.0051±0.0017	0.0091±0.0028	0.0056±0.0019	0.0053±0.0013	0.0048±0.0007
Average among rodent malaria	4	0.0248±0.0037	0.0240±0.0051	0.0525±0.0053	0.0397±0.0032	0.0418±0.0018
*P. falciparum*	101	0.0001±0.0001	0.0003±0.0001	0.0005±0.0003	0.0003±0.0001	0.0003±0.0001
*P. vivax*	110	0.0013±0.0005	0.0007±0.0005	0.0003±0.0001	0.0009±0.0003	0.0006±0.0001
*P. cynomolgi*	12	0.0036±0.0008	0.0033±0.0011	0.0032±0.0009	0.0034±0.0006	0.0026±0.0003
*P. inui*	14	0.0138±0.0017	0.0179±0.0029	0.0154±0.0022	0.0145±0.0015	0.0126±0.0011
*P. knowlesi* (all seq. together)	59	0.0013±0.0004	0.0016±0.0007	0.0008±0.0004	0.0011±0.0003	0.0009±0.0002
*Plasmodium* sp. from orangutan	11	0.0041±0.0008	0.0029±0.0009	0.0032±0.0007	0.0037±0.0006	0.0031±0.0003

**Figure 1:**
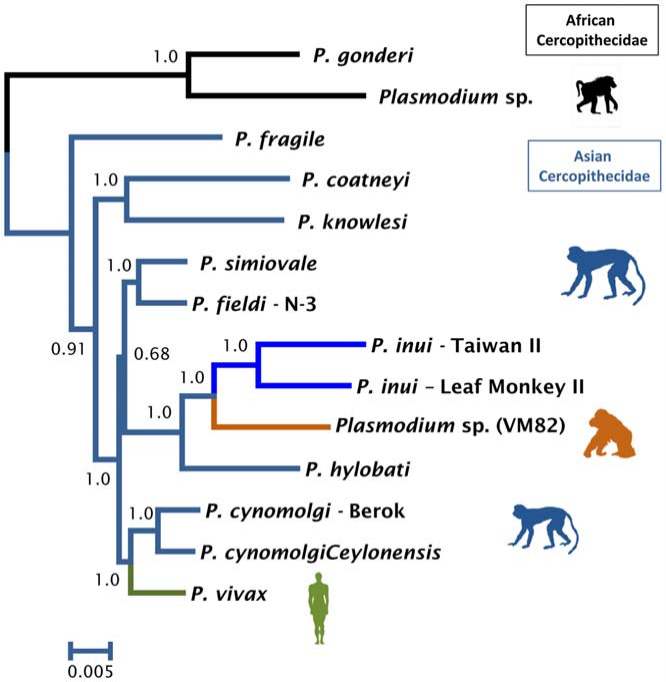
Phylogenetic tree of orangutan *Plasmodium* based on complete mitochondrial genomes. Bayesian and maximum likelihood methods yield identical topologies so only the Bayesian tree is shown. The values above branches are posterior probabilities together with bootstrap values (in bold) as a percentage obtained for the maximum likelihood tree (see methods).

**Figure 2:**
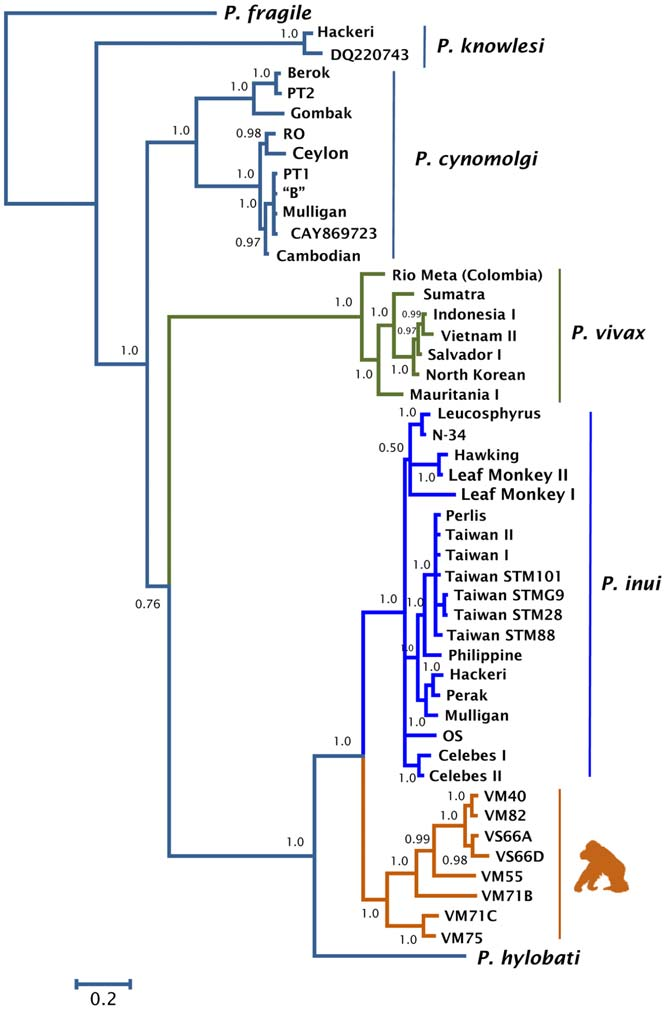
Phylogenetic tree of orangutan *Plasmodium* based on MSP1_42_ kDa. Bayesian and maximum likelihood methods yield identical topologies so only the Bayesian tree is shown. The values above branches are posterior probabilities together with bootstrap values (in bold) as a percentage obtained for the maximum likelihood tree (see methods).

**Figure 3:**
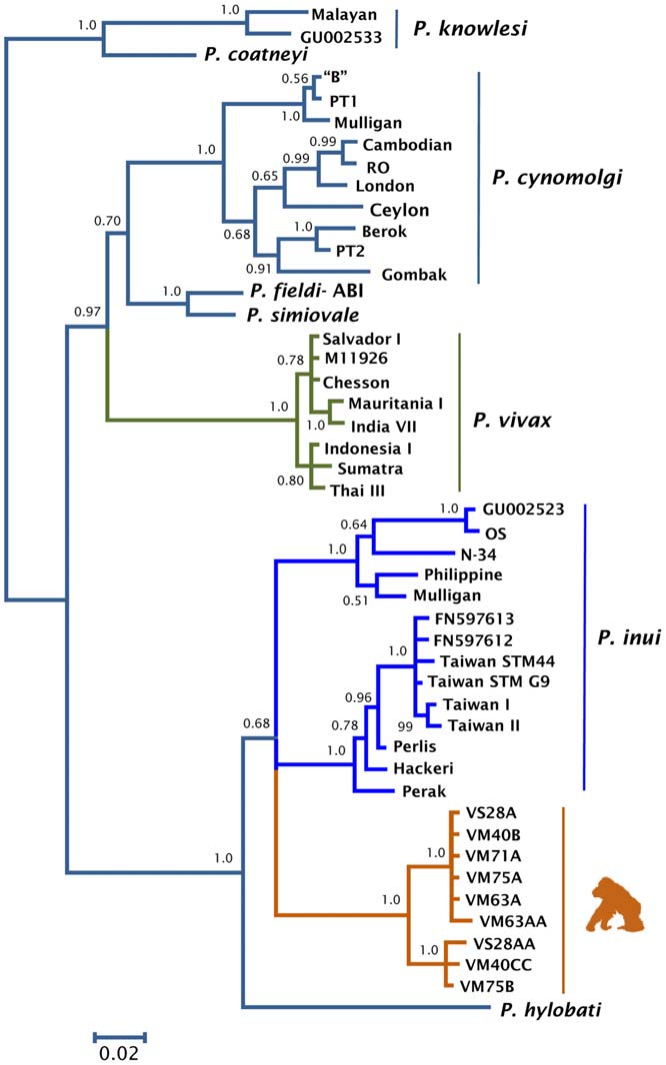
Phylogenetic tree of orangutan *Plasmodium* based on CSP gene (5′ and 3″ regions). Bayesian and maximum likelihood methods yield identical topologies so only the Bayesian tree is shown. The values above branches are posterior probabilities together with bootstrap values (in bold) as a percentage obtained for the maximum likelihood tree (see methods).

Phylogenetic analyses were performed separately on data from several non-human primate malarias and the orangutan parasite using complete mitochondrial genomes (Figure 1), MSP-1_42_ (Figure 2), and CSP (Figure 3). The phylogenetic analyses of the mitochondria and the MSP-1_42_ show that the orangutan malaria lineage is part of a clade sharing a recent common ancestor with *P. inui* (a macaque parasite) and *P. hylobati* (a gibbon parasite) (Fig. 1, 2). The CSP phylogeny, that only includes the non-repetitive region, does not allow to fully solve this relationship and there are two clades of *P. inui* with the orangutan clade forming a trichotomy.

The orangutan parasite alleles form a monophyletic group that is within the radiation of the primate malarias from Asia for the complete mitochondrial genome (Figure 1), MSP-1_42_ (Figure 2), and CSP (Figure 3); thus, this malaria parasite found in orangutans is not part of an ancestral lineage that could account for the radiation of primate malarial parasites in Southeast Asia. Given its derived position in the phylogeny, our findings indicate that this orangutan malaria lineage may have originated as a result of a host switch after the introduction of primate malarias in Asia by a non-ape host.

### Genetic polymorphism

In the case of the orangutan *Plasmodium* sp., the sequence encoding a fragment of the MSP-1 antigen, MSP-1_42_ [29] is a peptide of 375–382 amino acids in length, similar to *P. hylobati* MSP-1_42_ (381 aa) and other *Plasmodium* parasites (349–375aa) [30]. Table 2 describes the genetic variation found in MSP-1_42_, MSP-1_33_ and MSP-1_19_ [29] across the *Plasmodium* species. In contrast to MSP-1_42_ in *P. vivax*, where positive selection was observed [30], we found more synonymous than non-synonymous changes per site in orangutan *Plasmodium* sp. (Table 2). Overall, the pattern found in MSP-1_42_ in orangutan *Plasmodium* sp. and *P. cynomolgi* could indicate negative (purifying) selection acting on this protein [30]. The assumption of neutrality was also tested in most of the *Plasmodium* MSP-1_42_ orthologous by using the McDonald and Kreitman test [31] and there is evidence of negative selection in orangutan *Plasmodium* sp. (Table 3).

**Table 2:** Polymorphism found in the MSP1_42_ gene in different *Plasmodium* species.

MSP1	π (SE)	DS	DN	DS-DN (SE)	Z
***P. vivax*** (n = 75)					
42 kDa	0.0218	0.0125	0.0249	−0.0123(0.005)	Ds<Dn (*P*<0.05)
33 kDa	0.0325	0.0162	0.0325	−0.0160(0.006)	Ds<Dn (*P*<0.05)
19 kDa	0.0006	0.0005	0.0006	0.0001(0.000)	Ds = Dn
***P. cynomolg*** **i** (n = 10)					
42 kDa	0.0373 (0.0037)	0.0841	0.0283	0.0556 (0.0143)	Ds>Dn (P<0.05)
33 kDa	0.0408 (0.0043)	0.0952	0.0305	0.0647 (0.0173)	Ds>Dn (P<0.05)
19 kDa	0.0251 (0.0063)	0.0469	0.0211	0.0257 (0.0217)	Ds = Dn
***P. inui*** (n = 15)					
42 kDa	0.0240 (0.0025)	0.0271	0.0238	0.0033 (0.0057)	Ds = Dn
33 kDa	0.0289 (0.0030)	0.0068	0.0286	0.0049(0.0074)	Ds = Dn
19 kDa	0.0071 (0.0029)	0.0055	0.0077	−0.0022(0.0054)	Ds = Dn
***Plasmodium*** ** sp.** from orangutan (n = 8)					
42 kDa	0.0380 (0.0038)	0.0761	0.0296	0.0466 (0.0130)	Ds>Dn (P<0.05)
33 kDa	0.0445 (0.0041)	0.0859	0.0359	0.0500 (0.0160)	Ds>Dn (P<0.05)
19 kDa	0.0155 (0.0048)	0.0437	0.0088	0.0349 (0.0207)	Ds = Dn
***P. knowlesi*** (n = 2)					
42 kDa	0.0103 (0.0031)	0.0419	0.0024	0.0395 (0.0136)	Ds>Dn (P<0.05)
33 kDa	0.0109 (0.0035)	0.0427	0.0031	0.0396 (0.0154)	Ds>Dn (P<0.05)
19 kDa	0.0080 (0.0055)	0.0394	0	0.0394 (0.0282)	Ds = Dn

**Table 3:** McDonald and Kreitman test (neutrality index) for MSP1_42_.

	42 kDa	33 kDa	19 kDa
*Plasmodium* sp. vs. *P. vivax*	1.343 (NS)	1.343 (NS)	0
*Plasmodium* sp. vs. *P. cynomolgi*	0.425 (P<0.05)	0.425 (P<0.05)	0
*Plasmodium* sp. vs. *P. inui*	2.222 (NS)	2.222 (NS)	0

**Figure 4:**
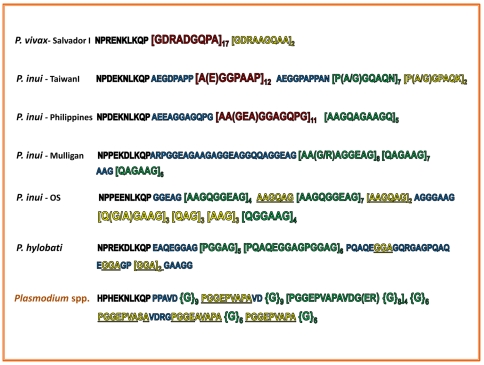
Amino acid composition of the Circumsporozoite protein tandem repeat sequences from *P. vivax*, *P. inui*, *P. hylobati* and *Plasmodium* spp. Repetitive motifs are reported between brackets “[]” followed by the number of times observed. Amino acids between parenthesis “()” are found in at least one of the motifs. The number of glycines observed in the orangutan lineage are indicated between braces “{}”. Color code and size indicate relative frequency: motifs found in tandem of 10 and more are indicated in red, motifs found in tandem of 4 to 9 are indicated in green, and motifs found in tandem from 2 to 4 are indicated in yellow. Blue sequences still show low complexity but not a discernible motif.

The second antigen studied is the gene encoding the CSP. In orangutan *Plasmodium* sp., like in other malarial parasites, it is a single 936–1140 bp exon gene encoding a 312–380 amino acid protein [11], [32]. As has been previously observed in the other *Plasmodium* species found in non-human primates [11], [32], the CSP protein shows high polymorphism in the number of repeats so only the non-repetitive region was used for phylogenetic analyses. The tandem repeat region in this orangutan lineage extends from 147 to 164 residues. The repeats in the orangutan *Plasmodium* sp. are clearly different from those found in *P. inui* and *P. hylobati* (Fig. 4). The first characteristic is the absence of the amino acid glutamine in the repeat regions, which is found in *P. hylobati* (10.2%) and *P. inui* (5–10% of residues). It is also relatively low in alanine, 16–19%, in contrast to 29–42.6% among the *P. inui* sequences and 22% in *P. hylobati*. However, like *P. inui* and *P. hylobati*, the tandem repeat regions are relatively rich in glycine (from 49 to 54% of the residues) (Fig. 4). Some of the motifs found in *P. inui* are also rich in the amino acid proline (28.6–30.4% of the residues); however, these were not observed in this *Plasmodium* sp. from orangutans. The motifs themselves are also divergent from those found in *P. inui* and *P. hylobati*, with the motifs EAV(V/A)P(G)_8–9_ and [PGGEPVAPAVDG(ER){G}_8_]_4_{G} (as an example see Fig. 4) among the most common ones in the orangutan parasite.

Table 4 describes the genetic variation found in the CSP gene, 5′ and 3′ regions across the *Plasmodium* species. The polymorphism found in the non-repetitive 5′ and 3′ regions together is lower than (π of 0.0134) the one reported for other parasites (e.g. π of 0.053 in *P. inui*). In order to determine if the observed polymorphism was under natural selection, we explored the number of synonymous (Ds) and the non-synonymous (Dn) substitutions in each species (Table 4). In general, we found that there are not significant differences between the number of synonymous and non-synonymous changes per site in the orangutan parasite (Table 4). The null hypothesis of neutrality was further tested in all *Plasmodium* CSP orthologous by using the McDonald and Kreitman test [31]. We found no evidence indicating that positive natural selection is acting on the CSP gene in the alleles of the orangutan *Plasmodium* sp. or any non-human primate malaria from Southeast Asia (data not shown).

**Table 4:** Polymorphism found in the CSP gene in different *Plasmodium* species.

CSP	π(SE)	DS	DN	DS-DN (SE)	Z
***P. vivax*** (n = 8)					
Gene	0.0064 (0.0022)	0.0060	0.0067	−0.0007 (0.0056)	Ds = Dn
5′ region	0.0063 (0.0036)	0	0.0083	−0.0083 (0.0068)	Ds = Dn
3′ region	0.0066 (0.0027)	0.0105	0.0056	0.0050 (0.0084)	Ds = Dn
***P. cynomolg*** **i** (n = 8)					
Gene	0.0385 (0.0054)	0.06675	0.0325	0.0343 (0.0173)	Ds = Dn
5′ region	0.0480 (0.0094)	0.0862	0.0399	0.0463 (0.0321)	Ds = Dn
3′ region	0.0316 (0.0067)	0.053	0.0271	0.0259 (0.0203)	Ds = Dn
***P. inui*** (n = 12)					
Gene	0.0490 (0.0063)	0.0486	0.0521	−0.0035 (0.0165)	Ds = Dn
5′ region	0.0451 (0.0070)	0.0396	0.0492	−0.0096 (0.0224)	Ds = Dn
3′ region	0.0518 (0.0082)	0.0557	0.0546	0.0011 (0.0299)	Ds = Dn
***Plasmodium*** ** sp.** from orangutan (n = 9)					
Gene	0.0134 (0.0032)	0.0182	0.0123	0.0059 (0.0088)	Ds = Dn
5′ region	0	0	0	0	Ds = Dn
3′ region	0.0232 (0.0056)	0.0321	0.0217	0.0104 (0.0016)	Ds = Dn
***P. knowlesi*** (n = 3)					
Gene	0.0080 (0.0032)	0.0180	0.0053	0.0127 (0.0107)	Ds = Dn
5′ region	0.0064 (0.0042)	0.0272	0	0.0271 (0.0187)	Ds = Dn
3′ region	0.0094 (0.0044)	0.0108	0.0092	0.0016 (0.0118)	Ds = Dn

### Estimation of divergence times: methods and calibration points

**Figure 5:**
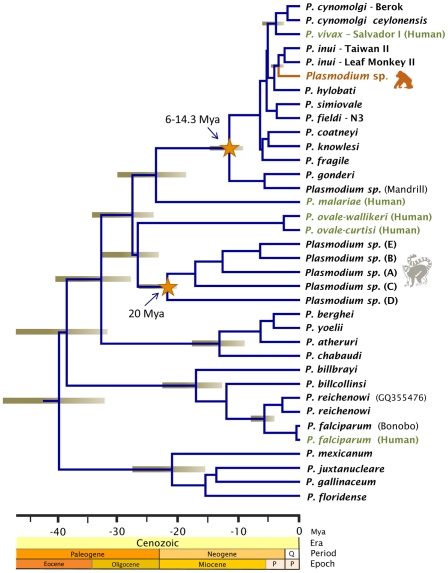
Timetree of the divergence of orangutan malarial parasites. Divergence times were estimated with BEAST using two calibrations points: the host divergence *Papio/Macaca* (6–14.3 Mya) and a minimum of 20 Mya for the origin of lemur parasites. Time is shown in millions of years ago (Mya).

A mitochondrial genome phylogeny that included other species of *Plasmodium* found in mammals was then used to estimate the time of origin of the orangutan lineage (Fig. 5). Table 5 shows *Plasmodium* divergence time estimates for two scenarios explored by using relaxed molecular clock methods that considered different assumptions. First, both scenarios shown in Table 5 (see Figure S1 in supporting information for the node numbers) assumed 20 Mya as minimum time for the introduction of lemur malarias into Madagascar [13], [33], [34]. Then, we explored two possible time intervals for the divergence of the African parasites found in Papionini from those found mostly in Asian macaques: one conservative that uses an interval of 6–8 Mya [24] narrowly defined on fossils and a second more inclusive estimate 6–14.3 Mya that also considers molecular time estimates [13] (see Table 5, Figure S1). As expected, the credibility intervals from both scenarios overlap. The times estimated using the inclusive calibration scenario, 6–14.3 Mya (Fig. 5), are 8 to 20% older with wider credibility intervals than those observed using the conservative calibration interval, 6–8 Mya. This effect is particularly noticeable in the Asian primate malarias, e.g. their origin (including *P. vivax*) is dated 5.4 Mya and 6.4 Mya in the conservative and inclusive scenarios respectively. The time estimates for the split between *P. inui* and the orangutan parasite reported here (2.85 Mya and 3.35 Mya for the conservative and inclusive scenarios respectively) are consistent with geological evidence indicating that several of the modern islands from insular Southeast Asia were actually connected by land that was exposed (Sundaland) due to lower sea levels [35], [36]. Such interconnected lands were populated by ancestral lineages of apes and macaque species that today harbor malarial parasites. It is worth noting that in both scenarios, the time estimated for the origin of the Catarrhini clade that includes *P. malariae* and *P. vivax* overlap with 23.5 Mya, the proposed minimum time for the divergence of *Macaca* and *Homo* [37].

**Table 5:** Divergence times of major splits in the malarial phylogeny as estimated by BEAST.

Calibrations		Node 13: min = 6, max = 8 Node 19: min = 20		Node 13: min = 6, max = 14.3 Node 19: min = 20	
Divergence	Node	Node Age (Mya)	95%CrI	Node Age (Mya)	95%CrI
Origin of Southerm Asia Primates malaria	11	5.42	4.38–6.63	6.43	5.01–8.24
Split *P. vivax - P. cynomolgi*	2	3.25	2.17–4.47	3.77	2.36–5.27
Split *P. inui - Plasmoium* sp. (from orangutan)	4	2.85	2.20–3.63	3.35	2.46–4.38
Origin of Catarrhini malaria (excluding *P. ovale*)	14	21.80	17.21–25.83	23.56	18.68–29.77
Lorisiforms-Catarrhini malaria	20	24.80	22.69–27.86	26.53	23.03–32.46
Radiation of African Apes malaria	30	15.07	11.32–19.27	16.94	12.58–22.40
Radiation of Rodents malaria	24	11.64	8.29–15.20	13.06	9.31–17.73
Split *P. falciparum - P. reichenowi*	28	5.05	3.60–6.87	5.72	3.95–7.89
Origin of *Plasmodium* in mammals	31	34.68	29.89–40.65	38.23	31.40–47.58

Calibrations, point time estimates and their associated 95% credibility intervals (CrIs) are shown in millions of years (Mya).

Node numbers are listed in Figure S1. See materials and methods section for more details.

## Discussion

The origin and radiation of human malarial parasites and their relationships with other *Plasmodium* sp. found in hominids have been the focus of attention for more than 50 years [16], [17]. Molecular phylogenies have been limited by the lack of representative samples from Asian apes, relying solely on the single available isolate from the gibbon parasite *P. hylobati* [2], [8].

Previous investigations suggested that orangutans may have acquired malaria from other primates in very recent times, at an ecological time scale or few generations ago [25], [28]. Whereas such is a biological plausible scenario, we found no evidence of a host switch at that time scale using more informative loci in our phylogenetic analyses. Indeed, the parasite that we describe is different to any previously identified *Plasmodium* species from which molecular data is currently available.

Given that the Miocene ancestors of orangutans and gibbons colonized Southeast Asia from western Eurasia [22]; this study starts to explore the possibility that these early apes were the original hosts for non-human primate malarias in Asia. However, our phylogenetic analyses indicate that the malarias found in Asian apes studied so far originated independently from those found in Africa, likely as the result of host switches from other Asian non-human primates.

Although classical parasitological investigations from the 30's to 70's did not include formal evolutionary analyses, they provided a reliable account of biological characteristics that allow for hypothesis testing using phylogenetic methods. The available morphological data indicated that the two described species, *P. silvaticum* and *P. pitheci*, share a recent common ancestor with *P. vivax* and related species [16]–[17]. Specifically, *P. pitheci* was considered “related” to *P. hylobati* whereas *P. silvaticum* was placed “closer” to *P. vivax* [17].

Given the lack of good morphological data, we cannot state whether the parasite lineage studied in this investigation belongs to any of the two species previously described or if it is a new one. However, this orangutan parasite shares recent common ancestor with *P. hylobati*, which agrees with some of the predictions made by classical parasitologists regarding *P. pitheci*. To be accurate, early descriptions of primate malarias did not consider any of the two *Plasmodium* sp. found in orangutans as particularly close to *P. inui* [16], [17]. It is worth noting that this orangutan parasite diverged only few million years ago from *P. inui* so short or slow evolving loci cannot properly separate them [25], [27]. Whereas short diagnostic sequences are widely used in field studies of malarial parasites (e.g. partial mitochondrial cytochrome b sequences); they may fail to capture the diversity of parasite lineages in primates [2], [9]. Since *P. inui* exhibit great diversity including a broad host range [18], [38]; hence, it will be important to ascertain the evolutionary history of what is currently recognized as *P. inui* since it could be indeed a species complex.

Our phylogenetic analyses using fast evolving gene encoding antigens have yielded phylogenetic relationships consistent with those derived from putatively neutral genes [11], [30]. Nevertheless, such genes encoding antigens appear to be under different selective pressures among non-human primate parasite lineages [30]. Thus, natural selection could be a source of rate heterogeneity among lineages, as well as evolutionary convergence, which could affect phylogenetic inferences. Such factors should be considered on a case by case basis whenever the aim is to ascertain species-level evolutionary relationships. In this particular study, it seems that selection by the host has not distorted the phylogenetic signal among these species since all the estimated phylogenies are consistent.

The combined use of fossil and biogeographical information as calibration points in molecular clock studies of malarial parasites seems promising. However, the limitations and consequences of assumptions need to be explored in each case [13]. By using mitochondrial genomes, we have access to a particularly rich database that allows for the exploration of different scenarios. In this study, the scenarios considered were internally consistent yielding credibility intervals that clearly overlap (Table 5, Figure S1). Our results indicate that this lineage of orangutan malaria originated at a time of lower sea levels when Borneo was connected with other parts of insular Southeast Asia by land bridges [35], [36]. Whether there were several host switches among Hominidea and non-Hominoidea primate malarias (e.g. parasites from macaques) are matters that cannot be addressed with the evidence presented here. It is worth noting that the split between *P. inui* and the orangutan parasite reported here (2.85 Mya and 3.35 Mya, Fig. 5), is consistent with early estimates for some of the mitochondrial haplotypes observed for the host orangutan sub-species *Pongo pygmeaus* [39], but is prior to the divergence between the two major orangutan sub-species [40]. Even more interesting, these time frames are consistent with the radiation of the genus *Hylobates*, the genus of the host harboring *P. hylobati* [41]. Our phylogenetic results suggest *P. inui* could represent a secondary acquisition of malaria in macaques from apes, with *P. hylobati* somewhat closer to the node of this clade. However, more information is still needed.

Given the extraordinary diversity of small apes in Southeast Asia, one can only speculate how important these primates may have been in the radiation of malarial parasites in the region. Unfortunately, out of the four species of *Plasmodium* described for gibbons, only a single isolate of *P. hylobati* is available.

An interesting observation is that the time estimate for the origin of the clade including *P. malariae*, *P. vivax*, and related parasites is consistent with *Homo-Macaca* split (minimum time 23.5 Mya, Fig. 5,) [37]. We explored the impact of considering such calibration point in molecular clock analyses and found the time estimates to be robust; that is, there are no major changes in the time estimates obtained by adding this calibration point (Table 5 versus Table 6, Figure S1). Indeed, including the 23.5 Mya point minimal calibration point for *P. malariae/P. vivax* and related species yielded estimates that are only slightly older (average 4–6%). Given that the inclusion of the *Homo*-*Macaca* split in calibrating the *P. malariae-*Asian primate clade does not change our conclusions, we suggest that this is a calibration point that could be considered in other investigations. Nevertheless, additional molecular clock investigations are needed that include nuclear genes in order to explore the effects of calibration points in timing malaria lineages.

**Table 6:** Divergence times of major splits in the malarial phylogeny as estimated by BEAST.

Calibrations		Node 13: min = 6, max = 8 Node 19: min = 20 Node 14: min = 23.5		Node 13: min = 6, max = 14.3 Node 19: min = 20 Node 14: min = 23.5	
Divergence	Node	Node Age (Mya)	95%CrI	Node Age (Mya)	95%CrI
Origin of Southerm Asia Primates malaria	11	5.69	4.50–6.93	6.66	5.19–8.30
Split *P. vivax - P. cynomolgi*	2	3.43	2.29–4.63	3.87	2.48–5.43
Split *P. inui - Plasmoium* sp. (from orangutan)	4	3.02	2.29–3.81	3.46	2.57–4.46
Origin of Catarrhini malaria (excluding *P. ovale*)	14	23.89	23.00–26.28	24.67	23.00–28.84
Lorisiforms-Catarrhini malaria	20	25.86	23.67–29.00	27.42	24.11–32.76
Radiation of African Apes malaria	30	15.61	11.71–20.13	17.46	12.78–22.64
Radiation of Rodents malaria	24	12.08	8.86–16.12	13.51	9.73–18.03
Split *P. falciparum - P. reichenowi*	28	5.28	3.68–7.09	5.93	4.13–8.16
Origin of *Plasmodium* in mammals	31	36.39	31.52–42.57	39.54	32.93–48.38

Calibrations, point time estimates and their associated 95% credibility intervals (CrIs) are shown in millions of years (Mya). Node numbers are listed in Figure S1. See materials and methods section for more details.

In conclusion, we report a unique, monophyletic lineage of malarial parasites found in orangutans. This lineage represents only the second parasite isolated from Asian apes. We found no evidence indicating that orangutans acquired Plasmodia from another host in recent times (ecological time scale or few generations ago); indeed, such a host switch took place approximately 3 Mya. Our molecular clock analyses suggest that the extant distribution of malarial parasites in Asian primates might have been determined by the complex geological processes that took place in insular Southeast Asia resulting in sea level changes [35], [36], a complex process that affected the extant distribution of their hosts. Our phylogenetic results do not contradict the current Asian origin model proposed for *P. vivax*. Additionally, our results provide support for the notion that hominids (humans, orangutans) acquired malarial parasites from non-hominid primates in Asia. Nevertheless, we also found evidence indicating that *P. inui* may have originated as a result of a host switch from apes into non-Hominidae primates. We strongly encourage further investigations on *Plasmodium* species in Asian apes, particularly those found in gibbons, since they are essential in elucidating the successful radiation of parasites in Southeast Asian primates and the chain of events leading to the origin of *P. vivax* as a human parasite.

## Materials and Methods

### Samples and ethic statement

Twenty four orangutan blood samples were collected in 2003 at the “Orangutan Care Center and Quarantine (OCC&Q)” in Borneo Indonesia under the permit U-675B-03 issued by the Simon Fraser University Animal Care Committee. The research was reviewed and approved by the Simon Fraser University Animal Care Committee under the Canadian Council on Animal Care guidelines. These samples are the same ones analyzed and previously published [25].

### Malaria molecular diagnostic

DNA was extracted from blood spots on Whatman® FTA® Classic Cards (Whatman Inc., Florham Park, NJ, USA) using QIAamp® DNA Micro Kit (Qiagen GmbH, Hilden, Germany). Each sample was screened for *Plasmodium* parasites by at least two independent PCR using primers for a 1200 bp fragment of the cytochrome b (cyt b) gene. Cytochrome b (cyt b) gene was chosen because it has been used widely in malaria ecology and evolutionary biology allowing us to access a relatively extensive dataset available in the GenBank (National Center for Biotechnology Information, National Institutes of Health) that includes *Plasmodium* and other haemosporidia parasites. The cytb primers used in this study were forward-TGT AAT GCC TAG ACG TAT TCC/reverse-GTC AAW CAA ACA TGA ATA TAG AC. PCR amplifications were carried out in a 50 µl volume reaction using 20 ng of total genomic DNA, 3 mM MgCl_2_, 1× PCR buffer, 1.25 mM of each deoxynucleoside triphosphate, 0.4 mM of each primer, and 0.03 U/µL AmpliTaq polymerase (Applied Biosystems, Roche-USA). The PCR conditions were: a partial denaturation at 94°C for 4 min and 35 cycles with 1 min at 94°C, 1 min at 53°C and 2 min extension at 72°C, a final extension of 10 min at 72°C was added in the last cycle. All the cytb fragments were identified as *Plasmodium* using BLAST showing significant similarity with different malarial parasite species found in primates (e.g. *P. inui and P. vivax*).

In order to establish the phylogenetic relationship between the parasites found in those orangutan samples and other primates *Plasmodium* species, for all positive samples, we amplified, cloned, and sequenced the complete mitochondrial genomes (mtDNA) and two antigens: the merozoite surface protein 1 42 kDa (MSP-1_42_) and the gene encoding the circumsporozoite protein gene (CSP). Details about the PCR, cloning and sequencing protocols are described below.

### Parasites mitochondrial genomes (mtDNA)

Approximately 5,800 bp of the parasites mitochondrial genomes (mtDNA) were amplified using the oligos Forward 5′ GAG GAT TCT CTC ACA CTT CAA TTC AAT TCG TAC TTC and Reverse 5′ CAG GAA AAT WAT AGA CCG AAC CTT GGA CTC with Takara LA Taq™ Polymerase (TaKaRa Takara Mirus Bio). The PCR conditions were: a partial denaturation at 94°C for 1 min and 30 cycles with 30 sec at 94°C and 7 min at 68°C, and a final extension of 10 min at 72°C. To detect mixed infections, samples were both cloned and direct sequenced. Mixed infections yield overlapping peaks in the sequence electropherogram when they are sequenced directly; they also can be evidenced by inconsistencies among haplotypes obtained by cloning from independent PCR amplifications and/or inconsistencies between sequences obtained by cloning and those obtained by direct sequencing (e.g. cyt b in this case). In all cases, at least two independent PCR products were cloned in the pGEM®-T Easy Vector Systems (Promega, USA), and four clones were sequenced from each individual. The mtDNA sequences reported in this investigation were deposited in GenBank under the accession numbers JQ308530–JQ308534.

### Merozoite Surface Protein 1 42 kDa (MSP-1_42_)

We compared the MSP1_42_ genes found in the orangutan samples against those sequences reported somewhere else [30], [42]. PCR was carried out in a 50 µl volume and included 20 ng/µl of total genomic DNA, 2.5 mM MgCl_2_, 1× PCR buffer, 1.25 mM of each deoxynucleoside triphosphate, 0.4 mM of each primer, and 0.03 U/µM of AmpliTaq Gold® DNA polymerase (Applied Biosystems, Roche-USA). The primer forward 5′ GAC CAA GTA ACA ACG GGA G and reverse 5′ CAA AGA GTG GCT CAG AAC C were used to amplify the MSP1_42_ gene, The PCR conditions were: a partial denaturation at 94°C for 4 min and 35 cycles of 1 min at 94°C, 1 min at 55°C, and 2 min extension at 72°C, and a final extension of 10 min was added in the last cycle. The amplified products were also purified, cloned using the pGEM® -T easy Vector Systems I from Promega (USA), and sequenced using an Applied Biosystems 3730 capillary sequencer. Both strands were sequenced from at least two or three clones. The MSP1_42_ sequences reported in this investigation were submitted to GenBank under the accession numbers JQ286394 to JQ286401.

### Circumsporozoite Protein gene (CSP)

To infer the history and processes in the evolution of the observed CSP polymorphisms, we use a set of different *Plasmodium* species from various geographic locations, thus increasing the probability of sampling the most divergent alleles. In the case of *P. vivax*, in addition to the Salvador I strain (XM_001616843) and Belem (M11926) available in the Genbank; we sequenced six laboratory isolates (Chesson from New Guinea, India VII, Indonesia I, Mauritania I, Sumatra, and Thailand III). Five sequences from different isolates of *P. cynomolgi* (strain B, Cambodian, PT1, PT2, and RO [18]), nine sequences of *P. inui* (Perak, Hackeri, Mulligan, N-34, OS from Malaysia, Perlis, and Philippine, Thai I and II [18]), one of *P. simiovale*, one of *P. fieldi* (ABI), one of *P. hylobati*, and one sequence of *P. coatneyi* were obtained in this study. All strains used were provided by the Center for Disease Control and Prevention. In addition, we included in our analyses some sequences previously reported for *P. cynomolgi* [32], *P. inui* (FN597612–13, FN597615, FN597620 [42] and GU002523 [43]), and two sequence of *P. knowlesi* available in the GenBank (Strain M [18]; and GU002533 [43]). In order to amplify the CSP gene, polymerase chain reaction (PCR) was carried out in a 50 µl volume and included 20 ng/µl of total genomic DNA, 2.5 mM MgCl_2_, 1× PCR buffer, 1.25 mM of each deoxynucleoside triphosphate, 0.4 mM of each primer, and 0.03 U/µM of AmpliTaq Gold ® DNA polymerase (Applied Biosystems, Roche-USA). The primer forward 5′ TAT ATA CMA GAA CAA GAT GAA G and reverse 5′ GGA TRT CAG CTA CTT AAT TG were used to amplify the complete CSP gene. The PCR conditions were: a partial denaturation at 94°C for 4 min and 35 cycles of 1 min at 94°C, 1 min at 52–54°C, and 2 min extension at 72°C, and a final extension of 10 min was added in the last cycle. The amplified products were purified, cloned using the pGEM® -T easy Vector Systems I from Promega (USA), and sequenced using an Applied Biosystems 3730 capillary sequencer. Both strands were sequenced from at least two or three clones. The CSP sequences reported in our study were submitted to GenBank under the accession numbers JQ308498 to JQ308529.

### Evolutionary genetic analyses

#### Phylogenetic analyses

Independent alignments for nucleotides sequences of mtDNA, MSP-1_42_, and the non-repetitive region of CSP were made using ClustalX version 2.0.12 with manual editing. Phylogenetic relationships were estimated using maximum likelihood (ML) methods as implemented in PhyML v 3.0 [44] and Bayesian methods using MrBayes v3.1.2 [45]. Both methods used a general time reversible+gamma model (GTR+−Γ) for mtDNA, and MSP-1_42_, and Kimura 2-parameter model for CSP, because these were the models with lower number of parameters that best fit the data as estimated by MEGA 5.0 [46]. The reliability of the nodes in the ML trees was assessed by bootstrap method with 200 pseudo-replications. Additionally, Bayesian phylogenetic analyses were performed as implemented in MrBayes [45]. In all Bayesian analyses, we used 3,000,000 Markov Chain Monte Carlo (MCMC) steps and discarded the 60% as a burn-in. Sampling was performed every 100 generations. Mixing of the chain and convergence was properly checked after each run.

#### Genetic polymorphism

For both CSP and MSP-1_42_ genes, we explored the genetic polymorphism within each species by using the parameter π, which estimates the average number of substitutions between any two sequences. In order to assess the effect of natural selection, we calculated the average number of synonymous (Ds) and non-synonymous substitutions (Dn) between a pair of sequences. The average number of Ds and Dn was estimated using Nei and Gojobori's method [46] with the Jukes and Cantor correction as implemented in MEGA 5.0 [46]. In addition, we estimated the difference between Ds and Dn, and the standard deviation was estimated by bootstrapping based on 1000 pseudo-replications for Ds and Dn, as well as a two tail Z-test on the difference between Ds and Dn [46]. The null hypothesis is that Ds = Dn; indicating the observed polymorphism was neutral.

The assumption of neutrality was also tested in both genes (CSP and MSP1_42_) for all the species included by using the McDonald and Kreitman test [31], which compares the intra- and inter-specific number of synonymous and non-synonymous sites. Significance was assessed using a Fishers exact test for the 2×2 contingency table as implemented in the DnaSP program version 5.10.01 [47]. In this analysis, we compared the *Plasmodium* sp. from orangutan samples against *P. inui*, the most closely related species found in this study.

#### Estimation of divergence times: methods and calibration points

We applied relaxed clock methods to estimate divergence times of malaria parasites. Times were obtained using BEAST v.1.6 on three mitochondrial genes (cox1, cox3, cytb) and a non-coding mitochondrial region. BEAST was chosen because it is widely used in timing analyses, is flexible, and its results are comparable with other studies [13]. Genes were treated as separate partitions and parameters were optimized specifically for each gene. Analyses were carried out using all four partitions (three genes and non-coding regions). The best fit model was (GTR+Γ), as found by MEGA 5.0, was used alongside a relaxed lognormal clock. Priors for the calibrations were described by uniform distributions so that no particular time point within a given time interval was favored. BEAST was run until convergence and good-mixing of the samples were reached [48]. In this investigation, we explored several scenarios that incorporate fossils and biogeographical landmarks as calibration points.

One the calibration points assumes that African parasites found in *Mandrillus* spp. and *Cercocebus* spp. [10], [14], [24] diverged from those *Plasmodium* spp. found in Southeast Asia macaques when *Macaca* branched from *Papio* [10], [14]. Fossils identified as *Macaca* spp. indicate that such an event took place 6–8 Mya as minimum boundaries [24]. The second calibration used is based on the colonization of Madagascar by terrestrial mammals, including lemurs. These colonization processes took place during a relatively short timeframe in the Cenozoic (∼65–20 Mya) [33], [34]. Thus, times younger than ∼20 Mya for the origin of lemur malarias are unlikely.

The first scenario explored used a conservative time period of 6–8 Mya narrowly defined around the fossils for the *Papio-Macaca* divergence with a minimum of 20 Mya for the origin of lemur parasites. The second scenario was a combination of the 6–14.3 Mya calibration with a minimum of 20 Mya for the origin of lemur parasites, where 14.3 Mya is the molecular estimate for the same *Papio-Macaca* divergence event [10], [13], [14]. The use of 14.3 Mya as a maximum considers also the fact that *P. gonderi* is a parasite of *Chlorocebus* (a Cercopithecini), making this scenario more inclusive [13]. Finally, we explored two additional scenarios in our discussion that used the human/*Macaca* split (23.5 Mya) as a calibration point. This possibility emerged after analyzing the first and second scenario and was used at the base of the primate malarias found in both Humans and Cercopithecoidea [37].

## Supporting Information

Figure S1 BEAST node numbers for the *Plasmodium* phylogeny as used in Table 5 and 6.
(TIF)
