# Supplementary figures and images for: The Origin of Malarial Parasites in Orangutans
Source: PLoS One. 2012 Apr 20;7(4):e34990. doi: 10.1371/journal.pone.0034990 (PMC3335055; doi:10.1371/journal.pone.0034990)

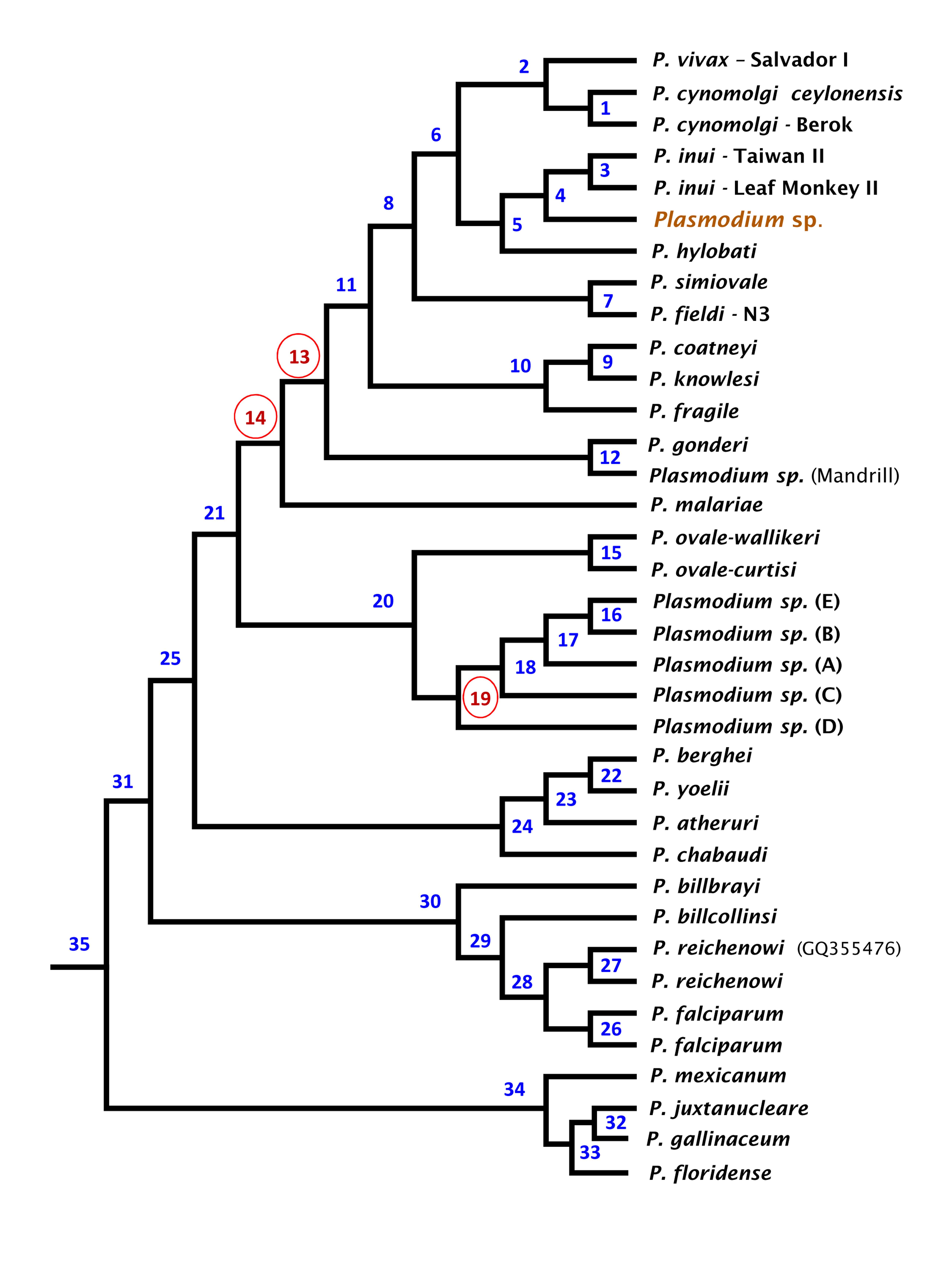

Supplement: Figure S1 — BEAST node numbers for the Plasmodium phylogeny as used in Table 5 and 6. (TIF) [file pone.0034990.s001.tif]
